# Investigating NF-κB signaling in lung fibroblasts in 2D and 3D culture systems

**DOI:** 10.1186/s12931-015-0302-7

**Published:** 2015-12-01

**Authors:** Su Su Htwe, Helen Harrington, Alan Knox, Felicity Rose, Jonathan Aylott, John W. Haycock, Amir M Ghaemmaghami

**Affiliations:** Cellular Immunology and Allergy Research Group, Division of Immunology, School of Life Science, Faculty of Medicine and Health Sciences, University of Nottingham, Nottingham, UK; Division of Respiratory Medicine, University of Nottingham, City Hospital, Nottingham, UK; Division of Drug Delivery and Tissue Engineering, Centre for Biomolecular Sciences, School of Pharmacy, University of Nottingham, Nottingham, UK; Laboratory of Biophysics and Surface Analysis, School of Pharmacy, University of Nottingham, Nottingham, UK; Department of Materials Science and Engineering, University of Sheffield, Sheffield, UK

**Keywords:** Nuclear factor Kappa B, NF-κB, Tumor Necrosis Factor-α, TNF-α, Lung fibroblasts, Lung inflammation, 3D cell culture, Electrospinning

## Abstract

**Background:**

Inflammatory respiratory diseases are amongst major global health challenges. Lung fibroblasts have been shown to play a key role in lung inflammatory responses. However, their exact role in initiation and maintenance of lung diseases has remained elusive partly due to the limited availability of physiologically relevant in vitro models. Therefore, developing new tools that enable investigating the molecular pathways (e.g. nuclear factor-kappa B (NF-κB) activation) that underpin inflammatory responses in fibroblasts could be a valuable resource for scientists working in this area of research.

**Results:**

In order to investigate NF-κB activation in response to pro-inflammatory stimuli in real-time, we first developed two detection systems based on nuclear localization of NF-κB by immunostaining and luciferase reporter assay system. Furthermore using electrospun porous scaffolds, with similar geometry to human lung extracellular matrix, we developed 3D cultures of lung fibroblasts allowing comparing NF-κB activation in response to pro-inflammatory stimuli (i.e. TNF-α) in 2D and 3D. Our data clearly show that the magnitude of NF-κB activation in 2D cultures is substantially higher than 3D cultures. However, unlike 2D cultures, cells in the 3D model remained responsive to TNF-α at higher concentrations. The more subdued and wider dynamic range of NF-κB responses in 3D culture system was associated with a different expression pattern for TNF receptor I in 3D versus 2D cultures collectively reflecting a more in vivo like TNF receptor I expression and NF-κB activation pattern in the 3D system.

**Conclusion:**

Our data suggest that lung fibroblasts are actively involved in the pathogenesis of lung inflammation by activation of NF-κB signaling pathway. The 3D culture detection system provides a sensitive and biologically relevant tool for investigating different pro-inflammatory events involving lung fibroblasts.

## Background

Inflammation is thought to be the central mechanism for many chronic lung disorders such as asthma, chronic obstructive pulmonary disease, and idiopathic lung fibrosis although the specific features and anatomical sites affected in each disease are different. Immune cells recruited at the site of inflammation are regarded as the key effector cells in lung inflammation through the production of various proinflammatory cytokines [1]. However, once inflammation is triggered, a chronic inflammatory response persists following lung injury. It is now accepted that in addition to immune cells, the structural lung cells such as airway epithelium, smooth muscles and lung fibroblasts also play an important role in initiation and maintenance of chronic lung inflammation through producing proinflammatory cytokines and growth factors [2–5].

Fibroblasts have been shown to produce a plethora of inflammatory mediators such as Interleukin-8 (IL-8), monocyte chemoattractant protein (MCP-1,3,4), macrophage inflammatory protein-1-alpha (MIP-1-α), eotaxin, granulocyte monocyte colony stimulating factor (GM-CSF), platelet derived growth factor (PDGF) and transforming growth factor-beta (TGF)-β during the acute and chronic phase of lung inflammation[6–9]. At the transcriptional level, the expression of genes controlling these inflammatory cytokines are under the control of nuclear factor kappa B (NF-κB), which plays a central role in regulating the expression of many genes involved in inflammation [10]. Fibroblasts can also proliferate and have the potential to transform into myofibroblasts [11, 12]. This could be in response to the cytokines produced by other cells (e.g. epithelial cell or monocytes) or through direct response to injury or exogenous stimuli [13, 14].

The ability to study the molecular pathways controlling the inflammatory responses in fibroblasts, such as those involved in NF-κB activation under in vivo like conditions will no doubt provide a better insight and understanding of lung inflammation, particularly in lung fibrosis where fibroblasts play a central role in pathogenesis. In addition to being a transcription factor controlling a number of inflammatory responses, NF-κB activation is probably the earliest cellular event in response to exogenous stress and injury [15]. Moreover, NF-κB has been shown to be activated in structural cells of lung in association with increased transcription of some proinflammatory cytokines and growth factors [16–18]. Thus, detection of NF-κB activation can not only act as a sensitive probe for inflammatory responses in human lung but also provide a useful tool for monitoring the impact of drug intervention or progression of chronic lung inflammation.

In unstimulated cells, NF-κB is bound to an inhibitor (IκB) in the cytoplasm preventing it from entering the nuclei. Upon exposure to external stimuli, IκB is promptly phosphorylated, ubiquitinated and degraded by 26S proteasomes releasing active NF-κB into the nucleus [19] in which it binds to a specific sequence in the promoter regions of target inflammatory genes leading to the activation of RNA polymerase type-II and synthesis of mRNA. Two cytokines, TNF-α and IL-1β, produced by inflammatory cells especially monocyte/macrophages in the early inflammatory stage, are also shown to be capable of setting up a positive feedback loop in NF-κB activation pathway which is important for perpetuation of a local inflammatory response [10]. Moreover these two cytokines have been shown to be involved in initial activation of fibroblasts for synthesis of inflammatory mediators [4, 7, 9, 20–22]. More importantly TNF-α itself has been demonstrated to play a central role in pathology of various pulmonary diseases [23].

Most studies on NF-κB activation have been performed in conventional 2D culture systems, however the limitations of 2D cell cultures are becoming more apparent [24, 25]. For example, in vivo fibroblasts are non-polar cells supported by ECM and exist in a 3D conformation within a particular geometry. The polarity, morphology and migratory properties of fibroblasts cultured in vitro on 2D surface becomes distorted in order to adapt the commonly used flat rigid artificial surface of tissue culture flasks/plates [26]. Human fibroblasts seeded in cell derived tissue matrix have higher proliferation than when cultured in 2D [27]. Moreover, it was shown that fibroblasts have greater cell attachment when cultured on 3D scaffolds, as they provide an environment for spatial organization, sustainment and high density culturing [27–31]. Furthermore, cells cultured in 3D rather than 2D are thought to be able to withstand more stress when encountered cytotoxic agents [24]. Thus, it is reasonable to assume that studying the fibroblast response to different stimuli leading to NF-κB activation in 3D better reflects the functional properties of these cells in vivo.

We have recently reported development of nano- and micro-diameter electrospun scaffolds from poly (ethylene terephthalate) (PET) capable of supporting the 3D growth and differentiation of lung epithelial cells and fibroblasts for up to 4 weeks [32, 33]. Interestingly, we and others have shown that such electrospun scaffolds with fibre diameters of approximately 240 ± 70 nm highly resemble the natural ECM of normal lung tissue (245 ± 83 nm) [33, 34].

Using such biomimetic electrospun scaffolds, in this study we investigated  NF-κB signaling in lung fibroblasts under inflammatory conditions using immunostaining and luciferase reporter assay that enable non-invasive monitoring of pro-inflammatory responses under in vivo like conditions.

## Methods

### Materials

Recombinant human TNF-α was purchased from Gibco (Life Technologies, UK) and Ready-To-Glow^TM^ NF-κB secreted luciferase reporter system (Cat No-631743) was obtained from Clontech laboratories (Palo Alto, CA). The Lipofectamine® 2000 (1 mg/mL) for transfection and Alamar Blue cell viability assay were obtained from Invitrogen (life technologies, UK). Fetal calf serum (FCS) was purchased from Labtech (UK) and Eagle’s minimal essential medium (MEM), L-Glutamine (200 mM), penicillin-streptomycin (10000 IU/mL/100 mg/mL) and trypsin-EDTA (10x) were from Sigma-Aldrich (UK). Primary anti-NF-κB/p65 (C-20) rabbit polyclonal IgG antibody was from Santa Cruz Biotechnology (sc-109, CA, USA), TNF receptor I rabbit polyclonal IgG antibody was from Abcam (19139) and Alexa Fluor® 488 goat anti-rabbit IgG secondary antibody (H + L) was from Molecular Probes (Life Technologies, UK).

### Polyethylene terephthalate (PET) scaffold fabrication

10 % Electrospun Polyethylene terephthalate (PET) was fabricated using published method described in Harrington *et al* [33]. Briefly, PET was dissolved in 1:1 trifluoroacetic acid (TFA): dichloromethane (DCM) (Fisher Chemicals, U.K.) to create a 10 % (w/v) solution. The polymer solution was loaded into a syringe (10 mL) with an 18 gauge needle (BD Falcon, U.K). The syringe was securely assembled on a syringe pump driver (Harvard Apparatus Ltd., U.K.). A grounded steel collector plate was positioned 15 cm distance from the needle tip. Then PET polymer solution was pumped at a constant flow rate of 0.5 mL/h at 14 kV for 4 h. Scaffold sheets were placed in a fume hood for 24 h to air dry. In order to have unique characteristics of scaffold such as thickness, diameter of fibres and pore sizes at each electrospinning, experimental conditions described in Table 1 were specified for each electrospinning. The pore size of the scaffold was determined using the SEM image analysis. According to Morris et al. [32], the pore size was defined by the longest distance within the defined pore on the same focal plane [32].

**Table 1:** Characteristics of PET scaffolds

Concentration of PET	Solvent (DCM:TFA)	Flow rate (ml/hr)	Needle Gauge	Voltage (kV)	Total Volume (ml)	Working Distance
10 % (w/v)	1:1	0.5ml/hr	18G	14kV	2ml	15cm

### Scanning electron microscopy

PET electrospun scaffolds were analysed by SEM (Scanning Electron Microscopy) (JEOL JMS-6060 LV microscope, U.K.) by mounting PET scaffold on carbon-coated electron microscope stubs and gold sputter coating for 4 min under an argon atmosphere (Blazers Union, SCD 030, BOC, U.K.) prior to analysis.

### Culture conditions and 2D cell culture

The human fetal lung fibroblast cell line (MRC-5) was obtained from the American Type Culture Collection (ATCC) and routinely cultured in T75 flasks in Eagle’s minimal essential medium (MEM) supplemented with 2 mM L-Glutamine, 10 % FCS and penicillin-streptomycin (100 IU/mL / 100 μg/mL) at 37 °C, 5 % CO_2_ in a humidified incubator until confluent. The culture medium was changed three times a week. When confluent, the cells were trypsinized with 1x trypsin-EDTA, centrifuged at 350 *g* for 5 min at room temperature, seeded directly into a 24 well plate (Costar) in transfection experiment and HCL treated coverslip was used for cell seeding in immunostaining. 1.5 ×10^5^ cells in 500 μL per well was used for all experiments. Then the cells were incubated in a humidified incubator for 24 h to allow cell adherence before transfection. Passage numbers 10–20 were used.

### 3D cell culture on PET scaffolds

Scaffold sheets were cut and sterilized under UV light (254 nm), 15 min on each side and then incubated with 70 % ethanol for 20 min in a non-tissue culture treated 12 well plates. Sterilized stainless steel rings (inner diameter - 1 cm, outer diameter - 1.9 cm and height - 0.8 cm) were used to hold down the scaffold and limit cell attachment in the centre of scaffold. After placing steel rings on the top of the PET scaffold, it was then sterilized with an antibiotic/antimycotic solution (5 %) (A5955, Sigma) overnight. Before cell seeding, PET scaffolds were washed with PBS x 2 times then soaked in complete MEM culture medium for 1 h. A cell suspension 1.5 × 10^5^ cells in 500 μL of complete MEM medium was seeded on the PET scaffold inside the steel ring while placing the same amount of medium outside. After seeding, the plate was incubated for 24 h to allow cell adherence to the scaffold before transfection.

### Immunostaining of NF-κB /p65 subunit and TNF receptor I expression (2D and 3D culture)

Briefly, the same culture condition for both 2D (coverslip) and 3D (PET scaffold) cultures was used for immunostaining of NF-κB and TNF-α receptor I expression. NF-κB activation was determined in MRC5 lung fibroblasts by immunostaining for the intracellular position of p65, the transcriptionally active subunit of NF-κB, as previously described [35]. Samples were fixed using 4 % (w/v) paraformaldehyde for 20 min at room temperature after 1 hr stimulation with TNF-α (2 ng/ml). TNF-α receptor I expression was studied without TNF-α stimulation and permeabilisation. Cells were permeabilized with 0.2 % (v/v) Triton for 20 min at room temperature and neutralized with 50 mM ammonium chloride for 5 min. Cells were washed three times in PBS after each treatment. Unreactive binding sites were blocked with 5 % (w/v) bovine serum albumin (Sigma) in PBS for 30 min. Cells were then incubated with primary anti-NF-κB/p65, anti TNF receptor I antibody for 1 h at room temperature (1:100 (v/v) in PBS). Cells were washed with PBS 3 times then incubated with Alexa Fluor® 488 goat anti-rabbit IgG secondary antibody (H + L) (1:1000 v/v in PBS) for 1 h at room temperature in the dark, washed 3x with PBS, followed by incubation with 300 nM DAPI (Sigma-Aldrich) in the dark for 30 min. Images were captured using 40x objectives of Zeiss LSM710 confocal with a Zeiss Observer microscope (SLIM imaging, University of Nottingham).

### Transfection of MRC5 lung fibroblasts (2D and 3D culture)

Transfection was performed as described in protocol provided from lipofectamine 2000® transfection agent. Briefly, the ratio 1: 2.5 of pNF-κB MetLuc2 reporter (μg) and transfection agent (μL) was used according to our optimization result. Firstly, reporter vector and transfection agent were diluted in 50 μL of Opti-MEM® reduced serum medium in separate tubes for 20 min at room temperature. Following which, both diluted reagents were mixed and incubated for 20 min at room temperature and then added 100uL dropwise into each well. Before addition, the culture medium was replaced with 400uL of Opti-MEM®. The cells were transfected for 5 h in incubators and then refreshed with complete MEM. In 3D culture, the *in situ* transfection method was used in our experiment and the same transfection procedure was followed as in 2D culture. One positive control with control vector was kept in both 2D and 3D experiments to determine transfection efficiency.

### Testing dose dependent TNF-α stimulation on NF-κB activation (2D and 3D culture)

Transfected MRC5 cells in both 2D and 3D culture were stimulated with 3 concentrations of TNF-α (0.2, 2 & 20 ng/mL) for 24 h in duplicate wells after 24 h transfection. Culture medium was refreshed 1 h before the experiment in order to reduce background reading. Then triplicates of 50 μL supernatants from each well were collected and assay was performed immediately according to the protocol provided (or samples were frozen at−20 °C until analysis). A white 96 well plate was then transferred to a luminometer (FLUOstar Optima) and analyzed. Data were recorded as relative light unit (RLU).

### Detection cell viability on 2D and 3D culture by Alamar Blue Assay

Following 24 h incubation with different doses of TNF-α, viability of MRC5 cells cultured in 2D and 3D was determined by Alamar Blue assay. Cells were incubated with Alamar blue dye (10 % of the volume of culture medium) for 4 h at 37 °C with protection from the direct light. 100uL of triplicate supernatants were transferred to a 96 black well plate (Fisher Scientific) and read at λex = 540 nm / λem = 580 nm (FLUOstar Optima)

### Data and statistical analysis

Statistical analysis was performed using Graph Prism 6, San Diego CA. Data are expressed as means ± SEM and analyzed by one way ANOVA with appropriate post hoc test for multiple comparisons. Significance level was set at *p* < 0.05.

## Results and discussion

### Development of 3D culture system using non-degradable Polyethylene terephthalate (PET) electrospun scaffold

**Fig. 1:**
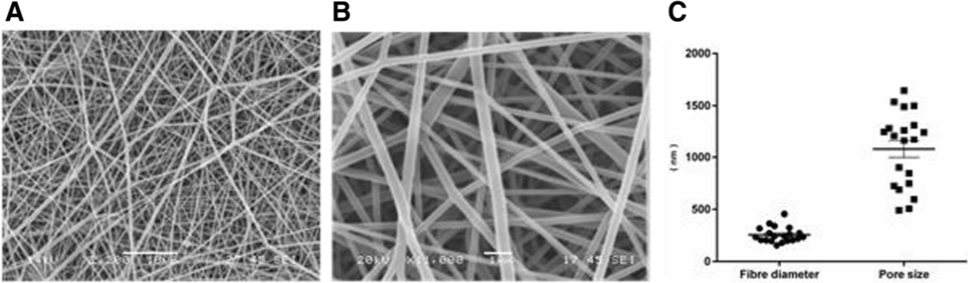
Acellular nanofiber PET scaffolds. Representative SEM images of 10 % PET acellular electrospun scaffold dissolved in 1:1 (v/v) DCM: TFA are shown in different magnifications **a** (Bar = 10 μm) and **b** (Bar = 1um). Distributions of fibre diameters and pore sizes of electrospun scaffold are shown in **c** (4 random measurements from 5 individual scaffolds) (Mean ± SEM, *n* = 20)

For developing the 3D cultures, we electrospun PET nanofibers (using the parameters summarized in Table 1) into fiber mats that were used for culturing fibroblasts in 3D. Scanning electron micrographs of PET scaffold confirms the random arrangement of nanofibers with non-beaded appearance (Figs. 1a and b). Fig. 1c shows average fibre diameter (257.4 ± 16.28 nm) and pore size (1079 ± 80.74 nm) (mean ± SEM) of 5 independent PET scaffolds. We have previously shown that these electrospun scaffolds have comparable geometry to human lung ECM and support fibroblasts growth and differentiation [32, 33]. Therefore, in line with our observations, fibroblasts grown on such scaffold are expected to maintain more in vivo like morphology, phenotype and functional properties compared to cells grown on 2D substrates .

### Detection of TNF-α induced NF-κB activation in lung fibroblasts using NF-κB/p65 nuclear translocation

**Fig. 2:**
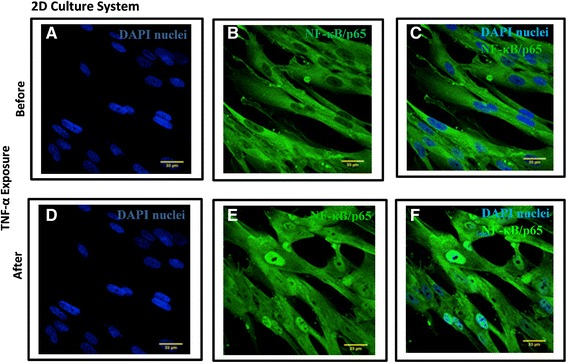
TNF-α induced NF-κB Activation in MRC5 lung fibroblasts in 2D culture system. Translocation of NF-κB/p65 was detected in MRC5 fibroblasts grown in 2D culture system after exposure to TNF-α (2 ng/mL) for 1 h by immunostaining (*green color*). Nuclei of lung fibroblasts were localized by DAPI (*blue*) (**a** and **d**). **a**–**c** represents cytoplasmic localization of NF-κB/p65 without TNF-α–treatment whereas **d**–**f** represents nuclear localization of NF-κB/p65 in MRC5 with TNF-α treatment for 1 h. The nuclear localization of NF-κB/p65 was overlaid by DAPI nuclear staining (**f**). (Bar =33 μm, *n* = 3)

**Fig. 3:**
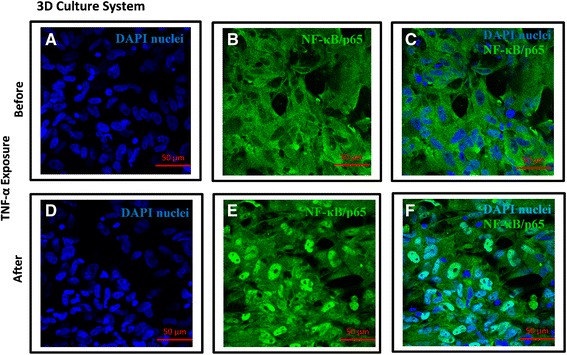
TNF-α induced NF-κB Activation in MRC5 lung fibroblasts in 3D culture system. Translocation of NF-κB/p65 was detected in MRC5 fibroblasts grown in 3D culture system after exposure to TNF-α (2 ng/mL) for 1 h by immunostaining (*green color*). Nuclei of lung fibroblasts were localized by DAPI (*blue*) (**a** and **d**). **a**–**c** represents cytoplasmic localization of NF-κB/p65 without TNF-α–treatment whereas **d**–**f** represents nuclear localization of NF-κB/p65 in MRC5 after TNF-α treatment for 1 h. The nuclear localization of NF-κB/p65 was overlaid by DAPI nuclear staining (**f**). (Bar =50 μm, *n* = 3)

Using fluorescent microscopy, we monitored nuclear localization of p65 subunit of NF-κB in both 2D and 3D cultures as a surrogate for NF-κB activation. Figures 2 and 3 show the NF-κB/p65 immunostained images of MRC5 lung fibroblasts grown on 2D and 3D culture, respectively, after 1 h stimulation with TNF-α (2 ng/mL). Data clearly show that NF-κB/p65 expression (green) remained cytoplasmic with almost no nuclear staining in unstimulated MRC5 cells (Figs. 2 and 3, upper panel) whereas there was substantial nuclear NF-κB/p65 staining (Figs. 2 and 3, lower panel) following TNF-α stimulation. DAPI-nuclei labeling (blue) was used to confirm the location of nucleus. This finding confirms NF-κB activation in lung fibroblasts in response to proinflammatory stimuli TNF-α. Previous in vivo studies in both human [36–38] and animal models [39–41] have shown NF-κB activation is associated with a number of inflammatory lung diseases (e.g. allergic asthma and chronic obstructive pulmonary diseases). Taken together, our results provide evidence for the active involvement of lung fibroblasts in pulmonary inflammatory responses.

In addition to NF-κB activation, a different morphology of fibroblasts was observed between 2D and 3D culture systems. Unlike cells grown in 2D which are aligned next to each other with limited cell-cell contact, fibroblasts grown in 3D have significantly higher cell-cell interaction forming networks (Figs. 2 and 3, right panel). There are also clear differences in the nuclear alignment between the two systems visualised after DAPI staining (Figs. 2 and 3, left panel) where cells in 2D cultures showed parallel nuclear alignment compared to a random nuclear arrangement in the 3D culture system. Given the similarities between the topography of the 3D fibrous scaffolds and the lung ECM [32, 33, 42, 43], we believe the cellular orientation observed on 3D scaffolds is likely to be closer to their arrangement in vivo .

### A luciferase reporter assay for real-time and non-invasive detection of NF-κB activation in lung fibroblasts in 2D and 3D cultures

**Fig. 4:**
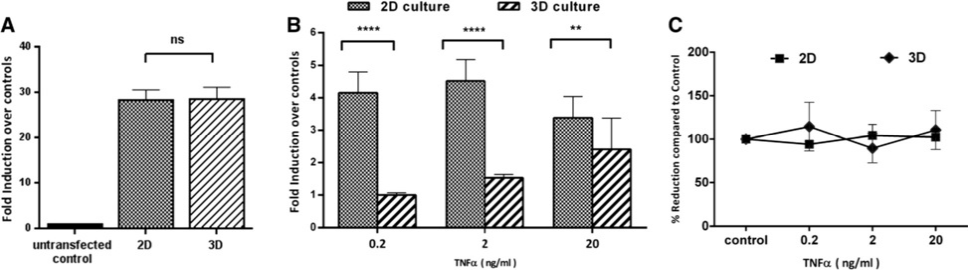
Detection of NF-κB activation in 2D and 3D cultures by luciferase reporter assay. **a** Comparison of transfection efficiency between 2D and 3D culture systems. Constitutive luciferase secretion in both systems was measured at 24 hr after transfection with control vector under same experimental conditions. Data expressed as fold induction over untransfected control value. **b** Comparison of NF-κB activation between 2D and 3D culture systems. Transfected MRC5 lung fibroblasts were stimulated with three different doses of TNF-α for 24 h in both systems after transfection. NF-κB activation was measured as fold induction increment of luciferase reporter activity from respective untreated control. **c** Assessment of cell viability between 2D and 3D culture system. Cell viability was assessed by Alamar Blue assay after 24 h stimulation with different doses of TNF-α. Untreated controls were defined as 100 % and experimental data were expressed as percentage reduction compared to that of control. Data are mean ± SEM of combined results from three independent experiments and collected as triplicates samples from duplicate wells for each experiment ****p < 0.0001,**p < 0.01, *ns = not significant*,(*n* = 3)

Having confirmed the NF-κB activation in lung fibroblasts by immunostaining, to better quantify such activation we used a luciferase reporter assay. In this method, NF-κB activation was monitored by the amount of luciferase protein secreted in the culture medium after TNF-α stimulation as the reporter construct expressing the luciferase gene is under the control of the NF-κB promoter element. Firstly both 2D and 3D cultures of fibroblasts were successfully transfected with a luciferase reporter vector after optimization of transfection efficiency with different ratios of transfection agent to control vector. To ensure parity between 2D and 3D cultures, we also investigated transfection efficiency between the two culture systems. To this end, the same number of lung fibroblasts were transfected with control vector which is designed to express luciferase protein constitutively without NF-κB promoter element. This was followed by culturing cells for 24 h in 2D or 3D before measuring luciferase activity. The 24 hr luciferase level after transfection was shown to be comparable between both systems (Fig. 4a).

To investigate the sensitivity of this assay for monitoring NF-κB activation, fibroblasts cultured in 2D and 3D were stimulated with three concentrations of TNF-α with 10 fold increments (0.2, 2 and 20 ng/ml) for 24 h (Fig. 4b). To assess any potential cell death after transfection and post TNF-α stimulation, we also determined the cell viability after 24 h exposure to different concentrations of TNF-α (Fig. 4c). This data showed no significant changes in cell viability between 3 concentrations of TNF-α in both 2D and 3D culture systems. Therefore any observed differences in NF-κB activation is not due to changes in cell viability.

As shown in Fig. 4b, all three concentrations of TNF-α significantly activated NF-κB dependent luciferase production in MRC5 fibroblasts compared to unstimulated control in both 2D and 3D cultures. However, 2D and 3D cultures showed a very different activation pattern and sensitivity to TNF-α stimulation. Despite the higher magnitude of NF-κB activation in 2D cultures, cells became non-responsive to TNF-α stimulation in concentrations above 2 ng/mL of TNF-α in contrast to 3D cultures in which cells remained responsive to TNF-α stimulation even at the highest concentration tested (i.e. 20 ng/mL). The drop in NF-κB activation at higher concentrations of TNF-α was not due to reduced cell viability in our experiment (Fig. 4c). Such ‘bell-shaped’ pattern of NF-κB activation in 2D cultures is in line with previous data showing a peak in NF-κB activation in HEK293 cells in response to 5 ng/mL TNF-α in 2D cultures followed by a drop at higher concentrations [44]. This effect could be explained by the negative feedback effect of newly synthesized IκBα at high level of NF-κB activation. In 2D cultures it has been shown that nuclear translocation of NF-κB activates the IκBα gene which has κB binding sites. Thus, the buildup IκBα can interfere with the binding of NF-κB to DNA by binding and bringing it back to cytoplasm. It can also prevent further NF-κB uptake by the nucleus [45, 46].

Interestingly, fibroblasts grown in 3D had a dose dependent response to all TNF-α concentrations albeit the level of NF-κB activation in 3D cultures was lower than 2D under the same experimental conditions (Fig. 4b). These observations are consistent with data from mouse lung tissue showing dose dependent but low level NF-κB activation in response to TNF-α compared response from liver tissue where NF-κB activation is at significantly higher levels but has a narrow dynamic range and drops with increasing concentrations of TNF-α [39]. Overall, these data suggest fibroblasts cultured in 3D exhibit a more in vivo like pattern of NF-κB activation in lung which is likely to be due to differences in TNF receptor expression or different regulatory mechanisms involved in controlling NF-κB activation in 2D versus 3D as a result of differences in cell-cell contact, cells morphology and polarization.

Together with immunostaining results, these data show that both detection methods are sensitive enough to detect NF-κB activation in lung fibroblasts cultured in 2D or 3D. However there were significant differences in the activation pattern and dynamic range of responsiveness to TNF-α stimulation in 2D and 3D culture systems.

### TNF receptor I expression on lung fibroblasts in 2D and 3D cultures

**Fig. 5:**
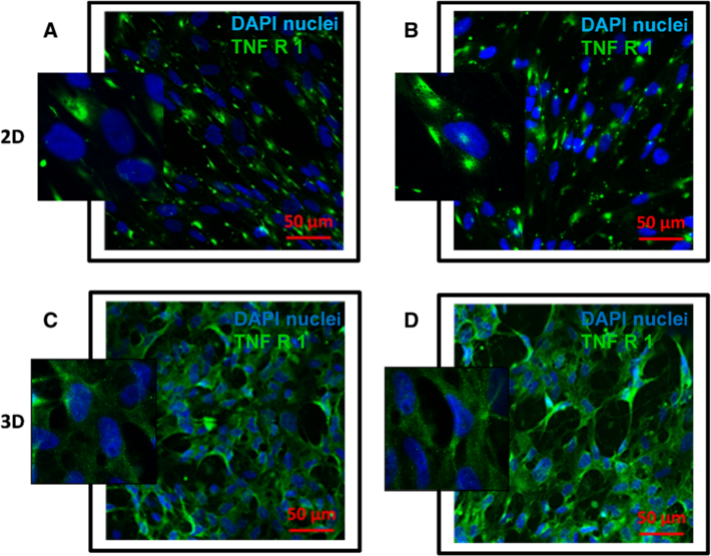
Immunostaining of TNF receptor I expression of lung fibroblasts in 2D and 3D culture systems. TNF-α receptor I on fibroblast surface was immunostained with Alexa Fluor® 488 secondary antibody (*green*) in both 2D (**a**–**b**) and 3D (**c**–**d**) culture systems and nuclei were labeled with DAPI (*blue*). (Bar =50 μm, *n* = 3)

Given that NF-κB activation in response to TNF-α is dependent on the surface expression of TNF-α receptor, it was reasonable to assume that the difference in the either pattern or level of TNF receptor expression between the two culture systems could drive difference in the NF-κB activation levels. Accordingly we examined the expression of TNF receptor type I (the main TNF receptor expressed on fibroblasts [47]) in cells cultured in 2D or 3D. Interestingly, receptor expression pattern was found to be different showing localized expression in 2D cultures (Figs. 5a and b) which was in contrast with homogenous expression pattern observed in 3D cultures (Figs. 5c and d).

As gap junction proteins can be expressed more in cell populations with higher cell to cell contact [48], we suggest that the formation of cellular networks in 3D culture system favours the formation of gap junctions between the fibroblasts. The exchange of molecules including ions and second messengers via these junctions may link to the homogenous receptor expression pattern in 3D system as compared to the focal expression pattern in 2D system [48, 49]. Such differential TNF receptor expression pattern between 2D and 3D culture systems could at least in part explain the different dynamics of NF-κB activation between two culture systems. Stromal cells such as fibroblasts naturally grow on 3D extracellular matrix bed that supports acquiring more physiologically relevant characteristics like receptor expression, proliferation and cytokine production [26]. Fibroblasts grown on 3D porous scaffolds achieved more complex morphology closer to the architecture of normal lung tissue [33, 42] and we propose that the observed NF-κB activation pattern in 3D culture is more in line with such responses in vivo. This is supported by studies in animal models showing dose dependent NF-κB activation in response to LPS challenge in lung inflammation of transgenic mouse model [39].

## Conclusions

In summary, we report the active involvement of lung fibroblasts in the context of lung inflammation by activating NF-κB signaling pathway. Moreover, the 3D culture system and NF-κB detection methods described in this study provide a simple but efficient tool for investigating proinflammatory responses non-invasively and in real-time under physiologically relevant conditions. To further enhance the physiological relevance of this model, the 3D fibroblast layer and the NF-κB detection system can be co-cultured with lung epithelial cells as well as relevant immune cells to create a multi cell immunocompetent 3D model of human lung. Such a model will provide a powerful tool for studying cellular and paracrine cross-talk between lung fibroblasts, immune cells and other structural cells in the context of acute and chronic lung inflammation.

## Abbreviations

NF-κB: Nuclear factor-kappa
TNF-α: Tumor necrosis factor- α
IκBα: Inhibitor of nuclear factor kappa B (NF-κB) alpha
2D: Two dimensional
3D: Three dimensional
PET: Polyethylene terephthalate
